# KOMPUTE: imputing summary statistics of missing phenotypes in high-throughput model organism data

**DOI:** 10.1093/bioadv/vbad100

**Published:** 2023-08-01

**Authors:** Coby Warkentin, Michael J O’Connell, Donghyung Lee

**Affiliations:** Department of Statistics, Miami University, Oxford, OH 45056, United States; InfoWorks, Inc., Nashville, TN 37205, United States; Department of Statistics, Miami University, Oxford, OH 45056, United States; Department of Statistics, Miami University, Oxford, OH 45056, United States

## Abstract

**Motivation:**

The International Mouse Phenotyping Consortium (IMPC) is striving to build a comprehensive functional catalog of mammalian protein-coding genes by systematically producing and phenotyping gene-knockout mice for almost every protein-coding gene in the mouse genome and by testing associations between gene loss-of-function and phenotype. To date, the IMPC has identified over 90 000 gene–phenotype associations, but many phenotypes have not yet been measured for each gene, resulting in largely incomplete data; ∼75.6% of association summary statistics are still missing in the latest IMPC summary statistics dataset (IMPC release version 16).

**Results:**

To overcome these challenges, we propose KOMPUTE, a novel method for imputing missing summary statistics in the IMPC dataset. Using conditional distribution properties of multivariate normal, KOMPUTE estimates the association Z-scores of unmeasured phenotypes for a particular gene as a conditional expectation given the Z-scores of measured phenotypes. Our evaluation of the method using simulated and real-world datasets demonstrates its superiority over the singular value decomposition matrix completion method in various scenarios.

**Availability and implementation:**

An R package for KOMPUTE is publicly available at https://github.com/statsleelab/kompute, along with usage examples and results for different phenotype domains at https://statsleelab.github.io/komputeExamples.

## 1 Introduction

**Figure 1.**
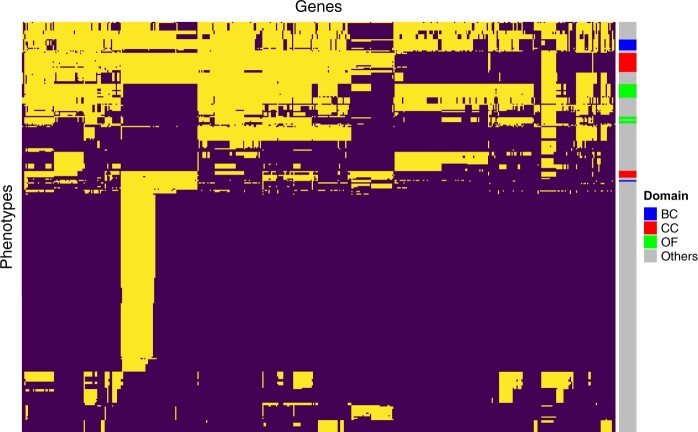
Heatmap visualization of data availability in the IMPC data (release version 16) for each gene–phenotype pair. The heatmap’s columns represent the 8216 genes studied via knockouts in mice, and the rows correspond to 303 distinct phenotypes. Dark purple cells indicate missing data, whereas light yellow cells indicate measured data. Binary clustering has been used to group together measured and unmeasured gene–phenotype pairs. The adjacent color bar denotes the phenotype domain each phenotype belongs to, with “BC” referring to Body Composition, “CC” to Clinical Chemistry, and “OF” to Open Field. “Others” represents other domains. Our subsequent analysis will focus on these three domains. Notably, only around 24.4% of the potential gene–phenotype pairs have been tested.

The International Mouse Phenotyping Consortium (IMPC) has been cataloging the functions of the entire mouse genome by producing knockout mouse lines for all protein-coding genes and examining the effects on various behavioral, physiological, morphological, and biochemical phenotypes (Meehan *et al.* 2017, Haselimashhadi *et al.* 2020). This process to date has successfully identified over 90 000 gene–phenotype associations in a controlled and reproducible setting (IMPC release version 16). However, generating a comprehensive functional catalog of the mouse genome is a daunting task, as there are many potential gene–phenotype pairs to consider, and testing all of them in a controlled setting is time-consuming and resource-intensive. Furthermore, knocking out vital genes can often result in early embryonic lethality or developmental abnormalities, making many phenotypes unmeasurable for mice from which these genes are removed (Dickinson *et al.* 2016). Therefore, many phenotypes of interest have not yet been measured for many genes, so the IMPC association summary statistics data are still largely incomplete (Fig. 1).

To recover summary statistics of missing phenotypes effectively, we propose a novel method, KOMPUTE. KOMPUTE leverages the conditional distribution properties of multivariate normal to directly impute association statistics (i.e. two-sided association Z-scores) of missing phenotypes, utilizing only the summary statistics of measured phenotypes and the corresponding phenotype correlations estimated as proxies for genetic correlations between phenotypes. A similar idea has been used to directly impute association Z-scores of unmeasured genetic variants in genome-wide association studies while maintaining high imputation accuracy and significantly reducing computational burden (Lee *et al.* 2013, 2015, Rueger *et al.* 2018). Similarly, the KOMPUTE method allows missing association Z-scores to be imputed directly, avoiding the intermediate step of imputing or measuring missing phenotypes for each mouse subject.

## 2 Methods

### 2.1 Summary statistics imputation

KOMPUTE estimates missing association Z-scores of phenotypes and genes using well-known conditional expectation formulas of the multivariate normal distribution (Lee *et al.* 2013). Assume that the summary statistics capturing the association between all phenotypes and genes form an m×n matrix of two-sided association Z-scores, where m is the total number of phenotypes observed, and n is the total number of genes tested. For a particular gene, let $Z_{1}$ be the k×1 vector of the unmeasured Z-scores (i.e. number of missing phenotypes = k) and let $Z_{2}$ be the l×1 vector of the measured Z-scores (i.e. number of measured phenotypes = m-k=l). By reordering phenotypes in this way, an m×1 column vector of Z-scores (i.e. the total number of phenotypes = m) for a gene can be written as


Z=Z1Z2.

Under the null hypothesis ($H_{0}$) of no association between gene-knockout and phenotype, Z asymptotically follows the multivariate normal distribution Z∼MVN(0,Σ), where Σ is the m×m variance–covariance matrix with unitary diagonal entries. Under $H_{0}$, Σ approximately equals the genetic correlation matrix between phenotypes, where the (*i*, *j*)-th entry represents the genetic correlation between phenotypes i and j, 0<i, j≤m. In the new ordering, Σ can be expressed as a block matrix as follows: where $\Sigma_{11}$ is the k×k genetic correlation matrix of unmeasured phenotypes, $\Sigma_{12}$ and $\Sigma_{21}$ are the k×l and l×k genetic correlation matrices between unmeasured and measured phenotypes, respectively, and $\Sigma_{22}$ is the l×l genetic correlation matrix of measured phenotypes.


$$\left[\begin{array}{cc} \Sigma_{11} & \Sigma_{12}\\ \Sigma_{21}\; & \;\Sigma_{22} \end{array}\right],$$

By using the conditional expectation formula of multivariate normal variates (Hogg *et al.* 2018), $Z_{1}$ (i.e. Z-scores of missing phenotypes) can be estimated as and the corresponding variance–covariance matrix is then estimated as


$$Z_{1|2}=\Sigma_{12}(\Sigma_{22}{)}^{-1}Z_{2},$$

$$\Sigma_{1|2}=\Sigma_{11}-\Sigma_{12}(\Sigma_{22}{)}^{-1}\Sigma_{21}.$$

To ensure that $\Sigma_{22}$ is invertible, a small ridge penalty (e.g. λ=0.01) can be added to each diagonal element of $\Sigma_{22}$. This ridge penalty is chosen to be small enough to have minimal impact on the imputed Z-scores (Lee *et al.* 2015). The diagonal of $\Sigma_{1|2}$ quantifies the uncertainty of the estimated $Z_{1|2}$ (Hogg *et al.* 2018). Therefore, the diagonal elements of $I-\Sigma_{1|2}$ can be used as an imputation accuracy measure (imputation information) of $Z_{1|2}$ (Lee *et al.* 2013). The imputation information value ranges from 0 to 1 for the corresponding imputed Z-score, with values closer to 1 indicating less variation in the imputed estimate and therefore a more reliable estimate.

By repeating this method for each gene in the original association summary statistics matrix, we can generate a new matrix with imputed Z-scores for the previously unmeasured phenotypes.

### 2.2 Estimating phenotypic correlation as a proxy for genetic correlation

Genetic correlation refers to the amount of variance shared between two phenotypes due to genetic factors. Estimating genetic correlations accurately can be difficult because it often requires collecting genetic information from very large samples, particularly when the heritability of the two phenotypes is low (Sodini *et al.* 2018). However, previous studies (Roff 1995, Reusch and Blanckenhorn 1998, Waitt and Levin 1998, Sodini *et al.* 2018) have found empirical evidence of a strong similarity between genetic and phenotypic correlations in insects, plants, animals, and humans when the sample size is large, based on the conjecture initially proposed by Cheverud (1988). Based on this evidence, we use the easier-to-estimate phenotypic correlation matrix observed in control mice ($\Sigma_{p}$) as a proxy for the genetic correlation matrix (Σ).

The raw phenotype data collected in experimental settings is often affected by nonbiological factors (e.g. phenotyping center) that can significantly impact measured phenotypes. In order to correct for these potential confounding factors, we use the principal variance component analysis (PVCA) (Li *et al.* 2009, Chen *et al.* 2011) to identify nonbiological covariates explaining a large proportion of phenotypic variation and Combat (Johnson *et al.* 2007) to remove the effects of these covariates from the phenotype data. The resulting adjusted phenotype data is then used to calculate the phenotypic correlation matrix ($\Sigma_{p}$) using the Pearson correlation coefficient. The estimated $\Sigma_{p}$ is used as an estimate for the genetic correlation matrix between phenotypes (Σ) in the imputation method (Section 2.1) (see Supplementary Fig. S1 for a detailed description of the process).

## 3 Results

To evaluate the performance of our proposed imputation method, KOMPUTE, we conducted extensive simulation studies and compared its performance to that of a singular value decomposition (SVD) matrix completion method (Kurucz *et al.* 2007). We simulated 100 association Z-score matrices (10 000 genes by eight phenotypes) from a multivariate normal distribution ${\mathrm{MVN}(0,\Sigma }_{P}$), where $\Sigma_{P}$ denotes the phenotype correlation matrix estimated from the eight body composition phenotypes of control mice.

We compared three different imputation methods: SVD matrix completion, KOMPUTE considering all imputed values, and KOMPUTE while only considering values with sufficiently high imputation information (above 0.8). Each of these methods was tested under three different scenarios where 20%, 40%, and 60% of the simulated Z-scores were randomly masked (i.e. temporarily deleting them from the data) and imputed. The Pearson correlation coefficient between masked Z-scores and imputed Z-scores was computed for each scenario and averaged across the 100 simulations.

The KOMPUTE method demonstrated superior performance compared to the SVD method across all simulation scenarios (Table 1). Furthermore, restricting our analysis to imputed Z-scores with high imputation information (i.e. KOMPUTE w/info > 0.8) resulted in even more reliable results. These highly accurate imputed values were obtained even when a significant proportion of the simulated data was missing.

**Table 1.** Pearson correlation coefficients between the original and imputed Z-scores.^a^

	20%	40%	60%
	Removed	Removed	Removed
SVD matrix completion	0.79	0.66	0.49
KOMPUTE	0.88	0.82	0.70
KOMPUTE w/info >0.8	0.95	0.95	0.96

a Three imputation methods were each tested under three different scenarios in which 20%, 40%, or 60% of the Z-scores were removed and imputed. The mean correlations over 100 simulations are presented, with a standard deviation <0.01 for all estimates.

In order to assess the performance of the proposed imputation method in realistic scenarios, we conducted additional experiments using real measured association Z-scores of three phenotype domains: body composition (8 phenotypes), clinical chemistry (19 phenotypes), and open field (14 phenotypes). For each domain, we first estimated a phenotype correlation matrix using control mice phenotypes, adjusting for nonbiological factors through PVCA and Combat (Supplementary Fig. S1). We then randomly masked 1000 gene–phenotype association Z-scores from each of the three domains and used the remaining Z-scores and the estimated phenotype correlation matrix to impute the missing values with the KOMPUTE method. We considered only the imputed Z-scores with high imputation information (>0.8) and calculated the Pearson correlation coefficient between imputed and original Z-scores as a measure of the effectiveness of KOMPUTE.

**Figure 2.**
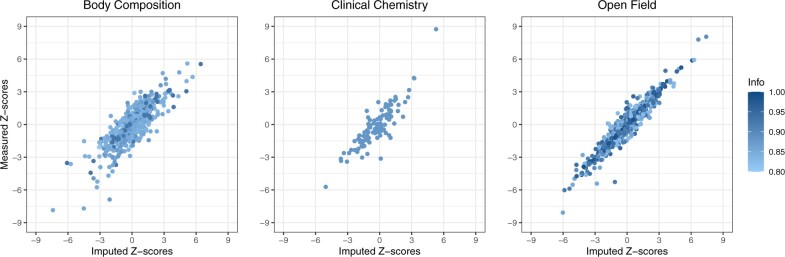
Measured Z-scores as a function of imputed Z-scores for three phenotype domains, namely body composition, clinical chemistry, and open field. “Info” in the legend title represents the imputation information value, serving as an indicator of the reliability of each imputed Z-score. A total of 1000 Z-scores were masked in each domain and imputed using the KOMPUTE method. To ensure the reliability of the imputed values, an imputation information score (Info) of at least 0.8 was required. The recovery rates for the body composition, clinical chemistry, and open field domains were 58.3%, 11.3%, and 84.6%, respectively, based on this threshold. The Pearson correlation coefficients between the original and imputed Z-scores for these domains were 0.84, 0.87, and 0.96, respectively.

Figure 2 shows the KOMPUTE method’s effectiveness in imputing missing Z-scores in realistic scenarios. In the body composition domain, all 1000 imputed Z-scores were complete cases with valid imputation information. The Pearson correlation between the original Z-scores and the imputed Z-scores was 0.79 (refer to the first row of Supplementary Fig. S2). Setting a cutoff for the imputation information at 0.8 to only include estimates with a higher degree of confidence resulted in 583 of the 1000 Z-scores being kept, with a Pearson correlation of 0.84 between these scores and the original masked Z-scores. For the clinical chemistry domain, all 1000 imputed Z-scores were complete and valid, but many of the imputation information values were relatively low, leading to a Pearson correlation of 0.49 between the masked and imputed Z-scores (refer to Supplementary Fig. S2). However, the 113 cases where the information was >0.8 had a Pearson correlation of 0.87 between the masked and imputed Z-scores. Finally, for the open field domain, all 1000 Z-scores were valid and generally had high imputation information values, resulting in a Pearson correlation of 0.93 between masked and imputed Z-scores (as shown in Supplementary Fig. S2). When the threshold for imputation information was set at 0.8, 846 out of the 1000 Z-scores met this criterion, and these imputed Z-scores showed a very strong Pearson correlation of 0.96 with the original masked Z-scores.

We further validated the effectiveness of KOMPUTE by comparing it with the SVD matrix completion method, using the same dataset across all three phenotype domains (as presented in the second row of Supplementary Fig. S2). In line with our simulation studies, KOMPUTE consistently outperformed the SVD method across all domains, highlighting its solid performance in imputing missing association summary statistics within high-throughput model organism data, under realistic conditions.

## 4 Discussion and conclusion

KOMPUTE has shown to be an effective method for imputing missing summary statistics in high-throughput model organism-based genetic association studies such as IMPC studies. The method outperformed the SVD matrix completion in all simulation scenarios and demonstrated good performance in more realistic settings. Furthermore, the use of an imputation information score allows for the identification of more reliable estimates. By improving the completeness of the IMPC dataset, KOMPUTE can help researchers unlock the full potential of this valuable resource. KOMPUTE also has the potential to be applied to a variety of high-throughput studies in various other model organisms, including *Caenorhabditis elegans* or *Arabidopsis thaliana*, where comprehensive phenotyping is a significant challenge.

High-throughput phenotyping in model organisms is typically conducted under stringent and controlled conditions, leading to the derivation of highly reliable phenotypic correlations and association Z-scores. This makes our KOMPUTE method, which relies heavily on Cheverud’s conjecture, particularly well-suited for this context. However, despite KOMPUTE’s demonstrated effectiveness, potential limitations exist. While Cheverud’s conjecture generally holds in controlled settings, there can be exceptions. Therefore, using $\Sigma_{p}$ as a surrogate for the genetic correlation matrix between phenotypes should be approached with caution. Additional validation is necessary when evidence indicates that the genetic correlation structure for a set of target phenotypes deviates significantly from the estimated $\Sigma_{p}$.

## Supplementary Material

vbad100_Supplementary_Data
