# Supplementary material for: KOMPUTE: imputing summary statistics of missing phenotypes in high-throughput model organism data
Source: Bioinform Adv. 2023 Aug 1;3(1):vbad100. doi: 10.1093/bioadv/vbad100 (PMC10409646; doi:10.1093/bioadv/vbad100)
Supplement: vbad100_Supplementary_Data [file vbad100_supplementary_data.pdf]

KOMPUTE: Imputing summary statistics of missing phenotypes in high-throughput model organism data

Supplementary Data

Coby Warkentin[1,2], Michael J. O'Connell[1] and Donghyung Lee[1,*]

[1]Department of Statistics, Miami University, Oxford, Ohio 45056, [2]InfoWorks, Inc., Nashville, Tennessee 37205

*Correspondence: leed13@miamioh.edu

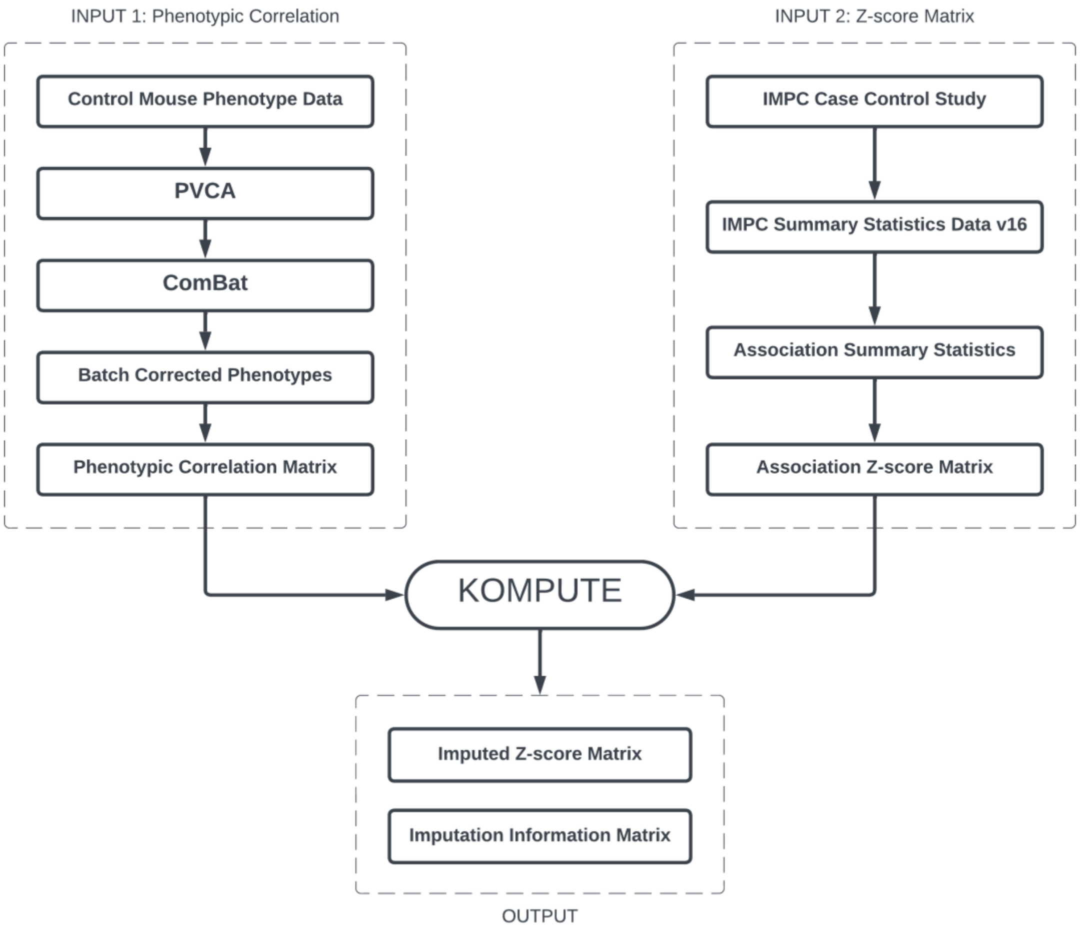

Figure S1. Schematic diagram illustrating the analysis process of the KOMPUTE method. KOMPUTE operates using two key input datasets: a phenotypic correlation matrix (**INPUT 1**) and an association Z-score matrix (**INPUT 2**). **INPUT 1:** The phenotypic correlation matrix is derived from measurements of hundreds of phenotypes initially gathered from over 30,000 control mice, where control mice represent the untreated group (i.e., without gene knockout). These data are normalized using a rank Z transformation. Subsequently, a Principal Variance Component Analysis (PVCA) is applied to identify significant non-genetic confounding variables (i.e., batch effect). If a batch effect is detected through PVCA, the ComBat method adjusts for this effect. The resultant phenotype data, now devoid of batch effects, is used to compute phenotype correlations and create a matrix. This matrix, acting as a proxy for the genetic correlation matrix, is utilized by KOMPUTE for the imputation process. **INPUT 2:** The association Z-score matrix is derived from the IMPC summary statistics (release version 16), which are obtained from the IMPC data repository. The data provides beta coefficient estimates and their standard errors for the genotype (i.e., Knockout vs Control). Association Z-scores are then calculated by dividing the beta coefficient estimate by its standard error. This data is reformatted into a wide-format matrix with genes and phenotypes represented by rows and columns, respectively. This matrix, which contains numerous missing Z-scores, serves as the primary input for KOMPUTE. **OUTPUT:** The KOMPUTE function returns two output datasets: an imputed Z-score matrix and a corresponding imputation information matrix.

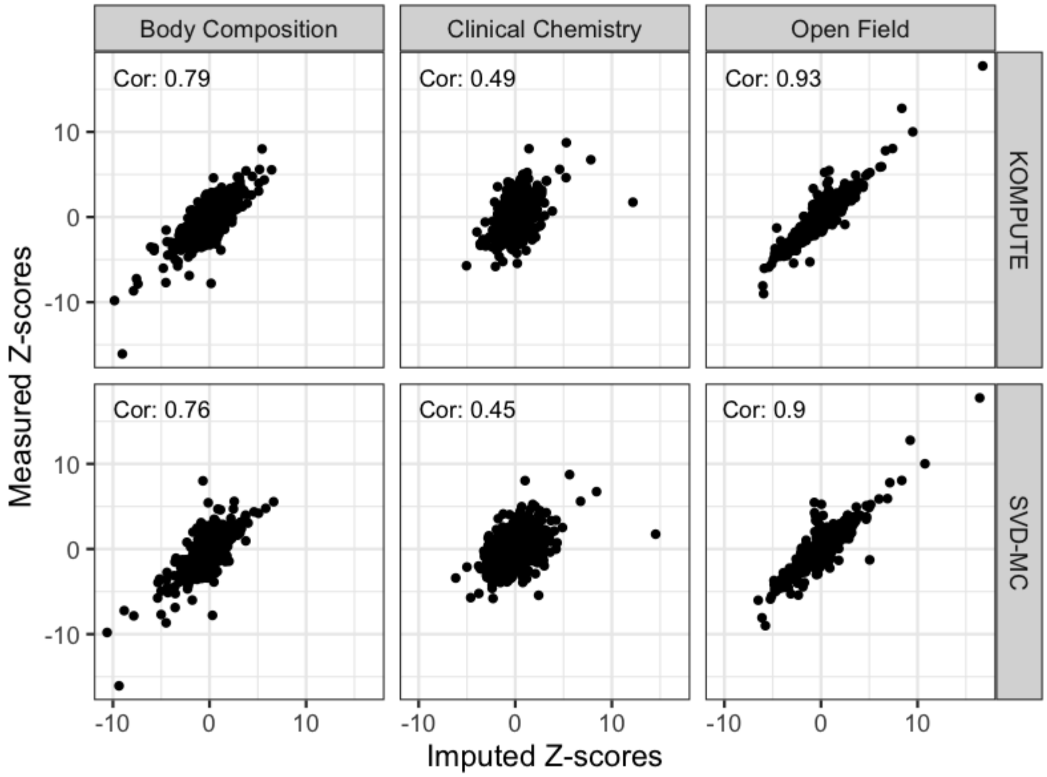

Figure S2. Comparison of imputed Z-scores to measured Z-scores across three phenotype domains (Body Composition, Clinical Chemistry, and Open Field) using two imputation methods (KOMPUTE and SVD Matrix Completion (SVD-MC)). Each plot represents the relationship between measured and imputed Z-scores for a given method-domain combination. The correlation coefficient (Cor) for each pairing is displayed in the top left corner of the corresponding figure. In this comparison, we include all imputed Z-scores, with no filters applied to exclude those with low imputation information. Across all domains, KOMPUTE consistently outperforms SVD-MC, highlighting its superior performance in imputing missing association summary statistics in high-throughput model organism data under realistic scenarios. Furthermore, KOMPUTE allows users to identify reliable imputed Z-scores easily using imputation information, as demonstrated in Figure 2.
